# Comprehensive pan-cancer analysis of PTGES3 and its prognostic role in hepatocellular carcinoma

**DOI:** 10.3389/fonc.2023.1158490

**Published:** 2023-05-18

**Authors:** Han Wang, Peng Sun, Ruoyu Yao, Wenrui Zhang, Xiaoshuang Zhou, Jia Yao, Kun He

**Affiliations:** ^1^ Department of Gastroenterology, Shanxi Bethune Hospital, Shanxi Academy of Medical Sciences, Tongji Shanxi, Hospital, Third Hospital of Shanxi Medical University, Taiyuan, China; ^2^ Department of Hepatobilary and Pancreatic Surgery, The Affiliated Hospital of Qingdao University, Qingdao, Shandong, China; ^3^ Department of Nephrology, The Affiliated People's Hospital of Shanxi Medical University, Taiyuan, China; ^4^ Department of Emergency Surgery, Shanxi Bethune Hospital, Shanxi Academy of Medical Sciences, Tongji Shanxi Hospital, Third Hospital of Shanxi Medical University, Taiyuan, China

**Keywords:** PTGES3, pan-cancer, hepatocellar carcinoma, immune infiltration, prognosis

## Abstract

**Introduction:**

PTGES3, also known as p23, is a molecule chaperone of Hsp90 that is involved in the pathogenesis of malignant tumors. Increasing studies have shown that PTGES3 plays a nonnegligible role in tumor development. However, analysis of PTGES3 in pan-cancer has not been performed yet.

**Methods:**

We explored the role of PTGES3 in 33 types of tumors and depicted the potentialimmune-related pathways among them. Using multiple databases includingTCGA, LinkedOmics, GDSC, and TIMER, we made a comprehensive analysis to explore whether there was an interaction between PTGES3 and prognosis, DNA methylation, copy number variation (CNV), tumor mutational burden (TMB), microsatellite instability (MSI), and tumor immune microenvironment (TME).

**Results:**

Our study revealed that PTGES3 expression level was upregulated in most cancers. PTGES3 was also associated with a positive or negative prognosis in a variety of cancers, which was mainly associated with DNA methylation, CNV, MSI, TMB, andmismatch repair-related genes. High PTGES3 expression was related to the infiltration of Th2 subsets of CD4+ T cells and immune checkpoint-related genes in most cancers, especially in hepatocellular carcinoma (HCC). Enrichment analysis demonstrated that PTGES3 was involved in cellular processes including DNA replication and spliceosome. The relationship between PTGES3 expression and HCC progression was verified at the protein level through immune histochemical analysis.

**Conclusions:**

Our research demonstrated theprognostic predictive value of PTGES3 in a wide range of cancers, which was alsoassociated with the process of tumor immune infiltration. As a result, it suggestedthat PTGES3 was a valuable prognostic biomarker in HCC treatment.

## 1 Introduction

Cancer remains a major health problem worldwide. Traditional treatments such as surgery, radiotherapy, and chemotherapy have their own limitations, and new treatments, such as molecular treatments, endocrine therapy, and immunotherapy, have attracted much attention. As a result, the exploration of novel biomarkers is urgent for the diagnosis, prognosis, and individualized therapy in cancer (1).

Prostaglandin E synthase enzyme 3 (PTGES3), also known as p23, is an oncogene mediating the expression of prostaglandin E2 (PGE2), which promotes tumor growth through multifactorial mechanisms such as upregulating anti-apoptotic genes (2). Furthermore, the COX/prostaglandin (COX/PG) pathway affects the progression of cancer mainly by the production of PGE2 (3). PTGES3 helps Hsp90 with protein folding and stabilizing in a wide range of proteins, which is proven to be involved in various biological processes and tumor pathogenesis (4). It is suggested that PTGES3 may be considered as a potential biomarker and therapy target. However, the role played by PTGES3 in cancer has only been reported in a few studies, and for only a few types of cancer. No bioinformatic analysis of PTGES3 in pan-cancer has been performed yet.

Therefore, we performed a comprehensive analysis of the association between PTGES3 expression and prognosis in 33 types of cancer via databases covering TCGA, LinkedOmics, GDSC, and TIMER. Furthermore, we investigated the potential genetic alteration of PTGES3 in various cancer types, which included copy number variation (CNV), DNA methylation, tumor mutational burden (TMB), and microsatellite instability (MSI). Immune infiltration levels of PTGES3 and the co-expression levels between PTGES3 and immune cell-related marker genes were also studied. Additionally, immunohistochemistry was used to confirm PTGES3 expression in HCC at the protein level, and the enrichment analysis was performed to probe the cellular functions of PTGES3 involved in HCC. Our study demonstrated that PTGES3 had an essential role in a wide range of cancers, indicating the prognostic potential of PTGES3 in hepatocellular carcinoma.

## 2 Materials and methods

### 2.1 Database source and expression analysis

UCSC Xena (https://xena.ucsc.edu/), a platform offering phenotype data of TCGA (https://tcga.xenahubs.net) to explore gene expression in cancer, was used to collect the RNA sequencing data, related survival and clinicopathological data, and somatic mutation data (5). The downloaded datasets were transformed using Strawberry Perl (Version 5.32.0, http://strawberryperl.com/) to evaluate PTGES3 expression in 33 tumors. The number of tumors of each type is shown in **Table S1**. The PTGES3 expression data were converted using the Log2 function, and two sets of *t*-tests were performed on each tumor type through the R software (Version 4.1.1). *p* < 0.05 was taken into account demonstrating differential expression between tumor and normal tissues. The results were presented via a boxplot using the “ggpubr” R package. We also compared the expression of PTGES3 in cancer samples and normal control using the TIMER2.0 database (6) as a supplement. Clinical proteomic tumor analysis consortium (CPTAC) (7) data were selected through the UALCAN database (http://ualcan.path.uab.edu/analysis-prot.html) to analyze PTGES3 expression at the protein level. Furthermore, we explored the relationships between PTGES3 and clinical phenotypes through the “Gene_ Outcome” module in TIMER 2.0. Clinical phenotypes such as tumor stage, gender, age, and race were selected to explore their association with PTGES3 expression.

### 2.2 Prognostic analysis of PTGES3 in pan-cancer

Overall survival (OS) and disease-specific survival (DSS) were selected to estimate the relationship between PTGES3 expression and prognosis in 33 types of cancer through Kaplan–Meier analyses and univariate Cox regression (uniCox) analyses with the R packages “survival” and “survminer”.

### 2.3 Methylation and CNV profile of PTGES3 in pan-cancer

Gene Set Cancer Analysis (GSCA) (http://bioinfo.life.hust.edu.cn/web/GSCA/) is a web server integrating multi-omics data from the TCGA database (8). The “mutation” module was selected for probing the connection between CNV, DNA methylation, and PTGES3 expression in pan-cancer.

### 2.4 Correlation between PTGES3 expression and TMB, MSI, and mismatch repair gene expression

TMB functions as a measurable immune-response factor indicating the amount of tumor cell mutations (9). MSI is brought on by MMR deficiencies that are associated with patient outcomes (10). Strawberry Perl was used to determine TMB scores in which the results were then adjusted through by dividing the sum of the exons. The somatic mutation data from TCGA were selected calculating the MSI scores of all samples through the link between TMB, MSI, and PTGES3 expression with Spearman’s rank correlation coefficient. The R packages “reshape2” and “RColorBrewer” were selected to generate the result in the form of heatmap. MMR was a cellular repair system for DNA. The abnormal expression of MMR-related genes was reported to lead to a higher frequency of somatic mutations with DNA replication errors that could not be repaired (11). The MMR genes’ expression in various cancers was assessed using expression profile data from TCGA. The correlation between PTGES3 expression and MMR-related genes was also determined. The results were visualized via “reshape2” and “RColorBrewer” R packages.

### 2.5 Immune infiltration analysis of PTGES3

Firstly, we calculated the level of immune cell and stromal infiltration of tumors using the Estimation of STromal and Immune cells in MAlignant Tumor tissues using Expression data (ESTIMATE) method with specific gene expression patterns (12). Then, the immune scores and stromal scores of tumors and their relationship with PTGES3 expression were put into calculation through the “estimate” and “limma” R packages.

Furthermore, the association between immune cell subset infiltration and PTGES3 in pan-cancer was analyzed through the TIMER2.0 database. The purity and infiltration level with log2 TPM as scale were illustrated by the “Immune Estimation” module of TIMER2.0. The *p*-values were obtained using Spearman’s correlation test.

In addition, we performed a co-expression analysis of PTGES3 and immune checkpoint-related genes using the R-package “limma”. Pearson correlation coefficients were calculated and the results were visualized using the “reshape2” and “RColorBreyer” R packages.

### 2.6 Drug sensitivity evaluation of PTGES3

The half-maximal inhibitory concentration (IC_50_) is the concentration necessary to suppress drug concentration by 50%. The correlation between PTGES3 expression and the IC_50_ of drugs in the GDSC database was explored in pan-cancer through the GSCA portal. We further divided samples of LIHC into a high-expression group and a low-expression group through the median expression of PTGES3 with the “limma” R package. The drug response of targeted therapy and chemotherapy for groups was examined using the “pRRophetic” R package and visualized in boxplot by the “ggplot2” R package. *p* < 0.05 was considered statistically significant.

### 2.7 Clinical factor analysis of PTGES3 in HCC

The correlation between PTGES3 expression and the clinical characteristics of HCC was analyzed, including pathologic stage, histologic grade, and tumor (T) stage. Meanwhile, univariate and multivariate Cox regression were carried out in order to further assess the prognostic value of PTGES3 in HCC patients. We also built a nomogram to explore the function of PTGES3 in the clinical prognosis of HCC, whose accuracy was verified by a calibration curve.

### 2.8 Gene enrichment analysis of HCC

Linkedomics (http://www.linkedomics.org/login.php) provided multi-omics data on tumors publicly, helping researchers to conduct tumor-related analytical research from multiple perspectives (13). We identified differential genes co-expressed with PTGES3 in HCC using the LinkFinder module, which were obtained via LinkFinder from the Firehose_RSEM_log2 (TCGA-LIHC cohort) dataset with Pearson’s correlation coefficient. Furthermore, these genes were visualized and subjected to Gene Ontology (GO) analysis and Genes and Genomes (KEGG) pathway enrichment analysis to investigate the potential functions of PTGES3 involved in the progression of HCC. Gene Set Variation Analysis (GSVA) of PTGES3 in HCC was also performed using the “GSVA” R package, in which the pathways significantly enriched were thought to be associated with PTGES3 expression. We choose “c2.cp.kegg.v7.1.symbols.gmt” as the reference gene set and *p* < 0.05 was considered statistically significant. The String database was selected to speculate the possible interaction network of PTGES3 (14).

### 2.9 Clinical sample and data collection

We collected 80 HCC tissues and paired non-cancerous tissues with all participants providing written informed consent. This research was approved by the Affiliated Hospital of Qingdao University Ethics Committee. The clinical data of these samples were also collected to verify the correlation between PTGES3 expression and clinical characteristics.

### 2.10 Immunohistochemistry analysis

Once resected, fresh tissue samples were obtained and fixed in 10% formalin. They were then placed on a glass slide, sliced into 3- to 4-μm-thick sections, and baked for 2 h at 60°C. The slides were then hydrated using an alcohol gradient and dewaxed with xylene. With 0.3% H_2_O_2_, the endogenous peroxidase activity was inhibited for 20 min. Following antigen retrieval, the slides were treated at 4°C for an overnight period with primary rabbit antihuman PTGES3 antibodies (1:500, ab133315, Abcam) before being incubated at 37°C for an additional 30 min with secondary anti-horseradish peroxidase antibodies. Hematoxylin-stained tissue slices were analyzed using 3,3′-diaminobenzidine (DAB). Finally, the pictures were collected using light microscopy following dehydration and sealing. We also determined the average optical density (mean density) of each image using ImageJ with blinding and compared the PTGES3 expression between HCC tissues and adjacent noncancerous tissues using an unpaired *t*-test.

### 2.11 Statistical analysis

The R 4.1.1 was used for statistical analysis. Two sets of *t*-tests were performed to identify differential expression between tumor and normal tissues on each tumor type. The Kaplan–Meier (KM) method and Cox regression analysis were implemented to assess the survival assays. Spearman or Pearson correlation analyses were performed to clarify the correlation between the groups. GraphPad Prism 8.0 software was used for experimental statistical analysis with unpaired *t*-tests. *p* < 0.05 was considered statistically significant.

## 3 Results

### 3.1 Basic expression of PTGES3 in pan-cancer

**Figure 1:**
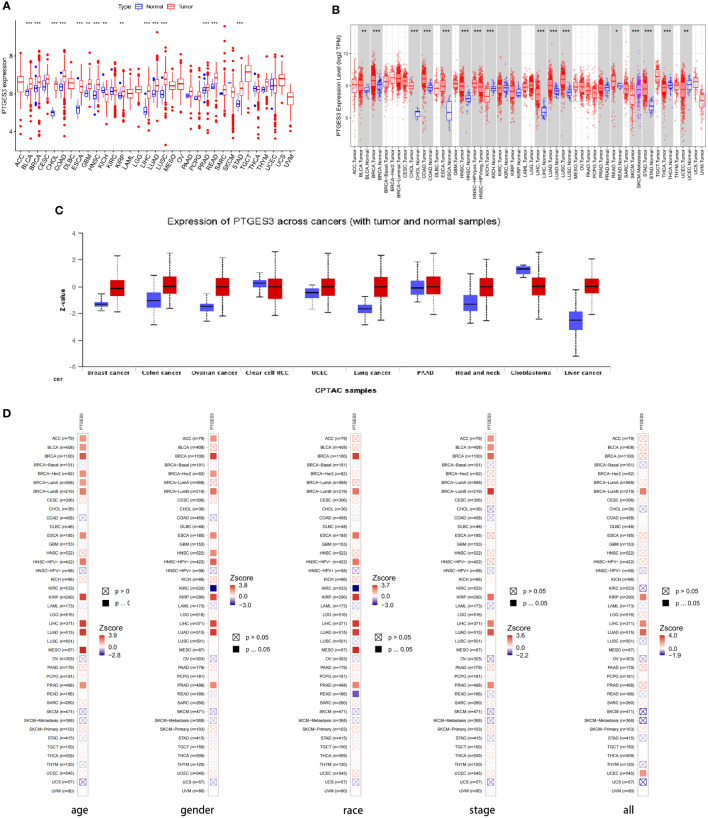
Differential expression of PTGES3. **(A)** PTGES3 expression in pan-cancer from the TCGA database. **(B)** TIMER2 analysis of PTGES3 expression in pancancer **(C)** Protein expression of PTGES3 from the CPTAC database. TIMER2 analysis of correlation between PTGES3 and clinical characters in pancancer, covering tumor stage **(D)** TIMER2 analysis of correlation between PTGES3 and clinical characters in pan-cancer, covering tumor stage, gender, age, and race. *p < 0.05; **p < 0.01; and ***p < 0.001.

We first analyzed PTGES3 expression in pan-cancer to determine the function of PTGES3 in carcinogenesis. By analyzing the obtained TCGA data, our results showed relatively high PTGES3 expression in 14 cancer types compared to normal tissues, covering liver colon adenocarcinoma (COAD), hepatocellular carcinoma (LIHC), lung squamous cell carcinoma (LUSC), esophageal carcinoma (ESCA), cholangiocarcinoma (CHOL), head and neck squamous cell carcinoma (HNSC), kidney renal papillary cell carcinoma (KIRP), lung adenocarcinoma (LUAD), breast invasive carcinoma (BRCA), glioblastoma multiforme (GBM), stomach adenocarcinoma (STAD), prostate adenocarcinoma (PRAD), bladder urothelial carcinoma (BLCA), and rectum adenocarcinoma (READ) (**Figure 1A**). We also found that PTGES3 was downregulated in kidney chromophobe (KICH). Furthermore, results in TIMER2.0 were similar to our results, which demonstrated differential expression in LIHC, ESCA, COAD, CHOL, HNSC, LUAD, LUSC, BRCA, STAD, READ, BLCA, and KICH (**Figure 1B**). The results of the CPTAC dataset indicated that the protein expression level of PTGES3 was significantly higher in tumor tissues than in normal tissues in COAD, BRCA, KIRC, OV, UCEC, LUSC, LIHC, and HNSC, but downregulated in GBM and no significance in PAAD (**Figure 1C**). The results in TIMER2.0 indicated that PTGES3 expression levels significantly correlated with clinical phenotypes of patients in BRCA, KIRP, LIHC, LUAD, and PRAD, which included tumor stage, race, gender, and age (**Figure 1D**). Furthermore, correlation analysis was performed using all these phenotypes adjusted with tumor purity, and the significant correlations were found in BRCA (Lumb), KIRP, LIHC, and LUAD.

### 3.2 Prognostic analysis of PTGES3

**Figure 2:**
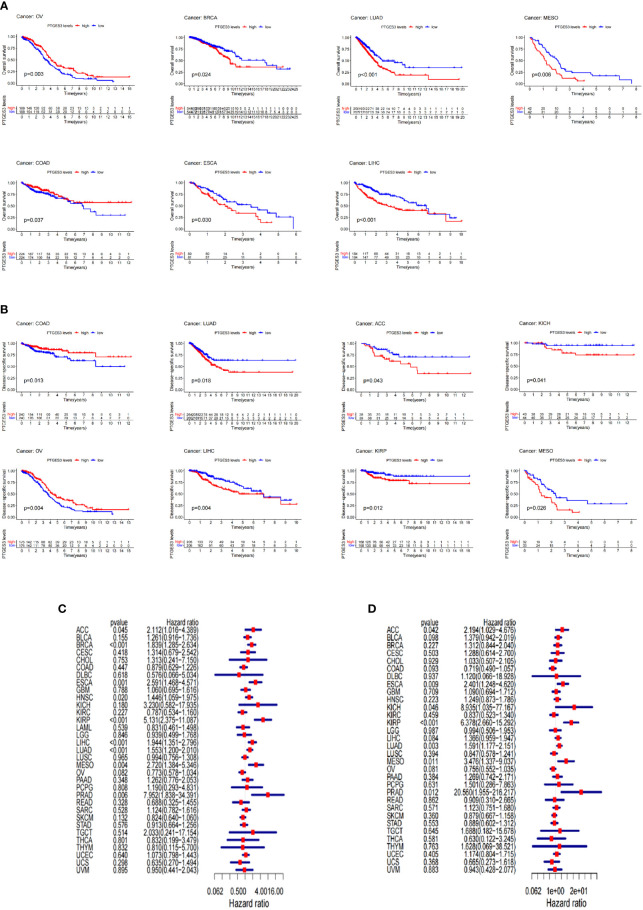
Survival landscape of PTGES3. **(A)** Significant relationship of PTGES3 expression and OS in *n* types of cancer based on Kaplan–Meier analysis. **(B)** Significant relationship of PTGES3 expression and DSS in *n* types of cancer based on Kaplan–Meier analysis. **(C)** Correlation between PTGES3 expression and OS based on univariate Cox regression analysis **(D)** Correlation between PTGES3 expression and DSS based on univariate Cox regression analysis. OS, overall survival; DSS, disease-specific survival.

The survival association for pan-cancer was processed to explore the association between PTGES3 expression level and prognosis of cancer patients. Our Kaplan–Meier survival results indicated that the relatively high PTGES3 expression was associated with poor OS in patients with BRCA, MESO, KIRP, LIHC, ESCA, and LUAD, while higher PTGES3 expression in patients with COAD and OV had a better OS (**Figure 2A**). The role played by PTGES3 in the DSS of tumor patients was observed for further evaluation (**Figure 2B**). The results revealed a correlation between relatively high PTGES3 expression and poor DSS in LUAD, ACC, LIHC, KIRP, MESO, and KICH, and a better DSS was also found in patients with COAD and OV. In addition, our Cox proportional hazards model analysis demonstrated that PTGES3 expression was associated with OS in BRCA, ESCA, HNSC, ACC, KIRP, LIHC, LUAD, MESO, and PRAD, particularly KIRP (hazard ratio = 5.131) (**Figure 2C**). It also revealed the associations between high PTGES3 expression and poor DSS in ACC, ESCA, KICH, KIRP, LUAD, MESO, and PRAD patients (**Figure 2D**). Our results indicated that PTGES3 expression might have a prognostic value in a wide range of cancer, especially LIHC, ESCA, MESO, and LUAD.

### 3.3 Methylation and CNV profile of PTGES3 in pan-cancer

**Figure 3:**
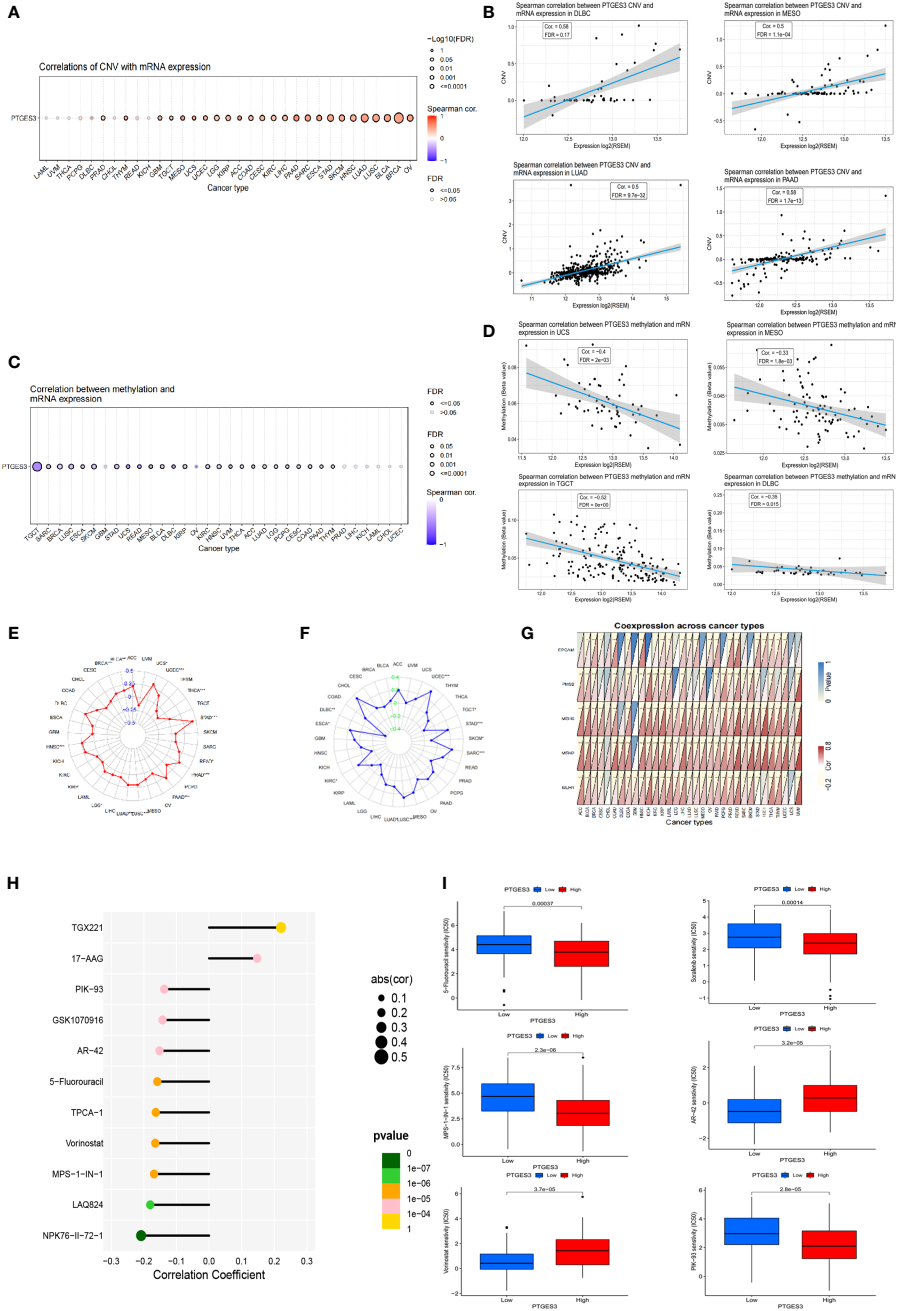
Genetic alteration, DNA modification, and drug sensitivity of PTGES3 in pan-cancer. **(A)** Correlation between PTGES3 expression and CNV in pan-cancer obtained from the GSCA platform. **(B)** Feature plot demonstrating the most correlated cancer types in CNV. **(C)** Correlation between PTGES3 expression and DNA methylation in pan-cancer obtained from the GSCA platform. **(D)** Feature plot demonstrating the most correlated cancer types in DNA methylation. **(E)** Relationship between PTGES3 expression and tumor mutational burden (TMB). **(F)** Correlation between PTGES3 expression and microsatellite instability (MSI). **(G)** Correlation between PTGES3 expression and five MMR-related genes in 33 types of cancer. **(H)** The most sensitive drugs of PTGES3 in pan-cancer via the GDSC database. **(I)** Box plot showing the drugs sensitive to PTGES3 expression in hepatocellular carcinoma (HCC). CNV, copy number variations; MMR, mismatch repair. *p < 0.05; **p < 0.01; and ***p < 0.001.

Using the GSCA database, the correlation between PTGES3 expression and CNV was investigated. In HNSC, BLCA, OV, LUSC, and LUAD, PTGES3 expression was substantially correlated with CNV (**Figure 3A**). Adversely, there was no significant correlation in diffuse large B-cell lymphoma (DLBC), PCPG, THCA, LAML, UVM, CHOL, READ, and KICH. The highest correlated tumor types were demonstrated (**Figure 3B**). We also explored the PTGES3 methylation landscape in pan-cancer (**Figure 3C**). It showed that PTGES3 methylation was strongly associated with PTGES3 expression in most cancer types except GBM, PRAD, LIHC, KICH, LAML, CHOL, and UCEC. The most correlated types such as TGCT, LUSC, and BRCA were demonstrated (**Figure 3D**).

### 3.4 Correlation analysis of PTGES3 with TMB, MSI, and mismatch repair genes

We investigated the correlations between TMB, MSI, and PTGES3 expression in order to find whether PTGES3 had sensitive correlations with immune checkpoint inhibitors. Our results showed that PTGES3 expression was correlated with 12 types of tumor in TMB and 10 types of tumor in MSI (**Figures 3E, F**). In addition, the co-expression association between PTGES3 expression and MMR-related genes was analyzed, which included EPCAM, MSH6, MLH1, PMS2, and MSH2 (**Figure 3G**). The illustrated results indicated that MMR gene expression was substantially positively correlated with PTGES3 expression.

### 3.5 Analysis of PTGES3 drug sensitivity

Using the GSCA database, we obtained the relationship between drug IC_50_ and PTGES3 expression in the GDSC database in pan-cancer. The 11 drugs most significantly correlated was illustrated, in which both TGX221 and NPK76-II-72-1 had a correlation above 0.2 (**Figure 3H**). Subsequently, we further explored the drug sensitivity of PTGES3 in HCC by dividing the samples of LIHC into high-expression and low-expression groups according to PTGES3 expression. The results showed that 106 drugs were significantly different between the two groups (**Figure 3I**).

### 3.6 Analysis of PTGES3 expression in tumor immune infiltration

**Figure 4:**
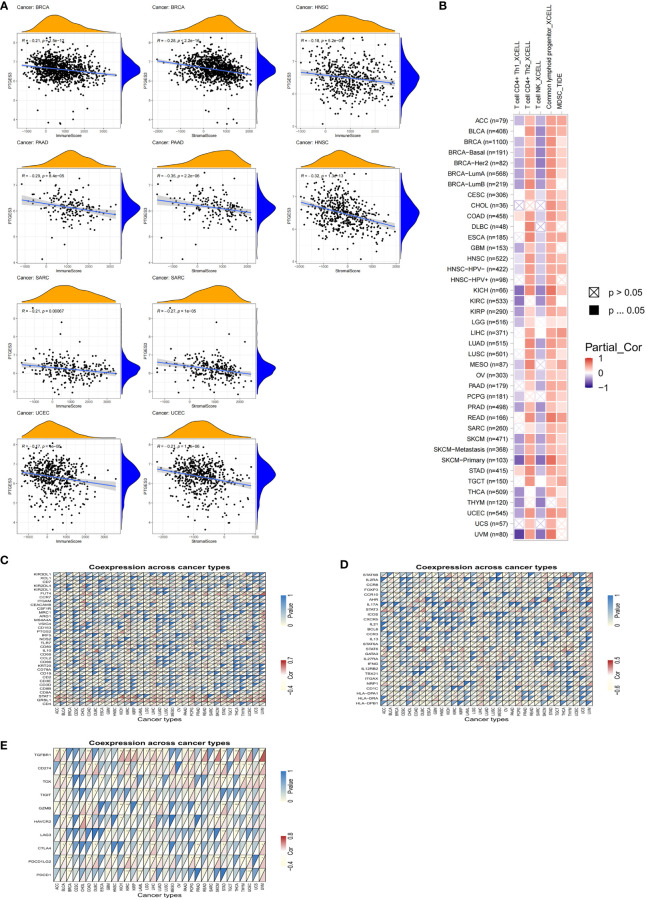
Relationship between PTGES3 expression and pan-cancer tumor immune infiltration. **(A)** Correlation between PTGES3 expression and the ESTIMATE score of the tumor microenvironment in pan-cancer. **(B)** The correlations of PTGES3 mRNA expression and immune cell infiltration across human cancers according to the TIMER2.0 database. **(C, D)** Relationship between PTGES3 expression and classic immune checkpoint-related genes. **(E)** Relationship between PTGES3 expression and specific immune cell marker genes. *p < 0.05; **p < 0.01; and ***p < 0.001.

Since PTGES3 expression had an association with TMB and MSI that affects the sensitivity of immunotherapy in multiple types of tumors, we explored the influence of PTGES3 expression on the abundance of tumor cell infiltrates. We firstly calculated the immune and stromal scores across human cancers via the ESTIMATE method. The scatter plot indicated that PTGES3 was correlated with BRCA, HNSC, PAAD, SARC, and UCEC in both scores significantly (**Figure 4A**). Meanwhile, the XCELL and TIDE algorithms were selected to explore the association of immune cell infiltration level and PTGES3 expression. The results demonstrated that PTGES3 was significantly negatively correlated with CD4^+^Th1 cells and NKT cells and positively correlated with CD4^+^Th2 cells, common lymphoid progenitors, and myeloid-derived suppressor cells (MDSCs) of various tumors (**Figure 4B**). The co-expression analysis showed marker genes of immune cells having a significant positive correlation with PTGES3, remarkably in HCC (**Figures 4C, D**). In addition, we also looked for correlation analysis between common immune checkpoint-associated marker genes and PTGES3, finding that PDCD1, CTLA4, HAVCR2, and TIGIT, and the expression of other classical immunosuppressive genes showed significant correlation over a wide range of cancers including LIHC, suggesting that there was a potential regulation of PTGES3 expression in the immune function of HCC (**Figure 4E**).

### 3.7 Clinical correlation analysis of PTGES3 in HCC

**Figure 5:**
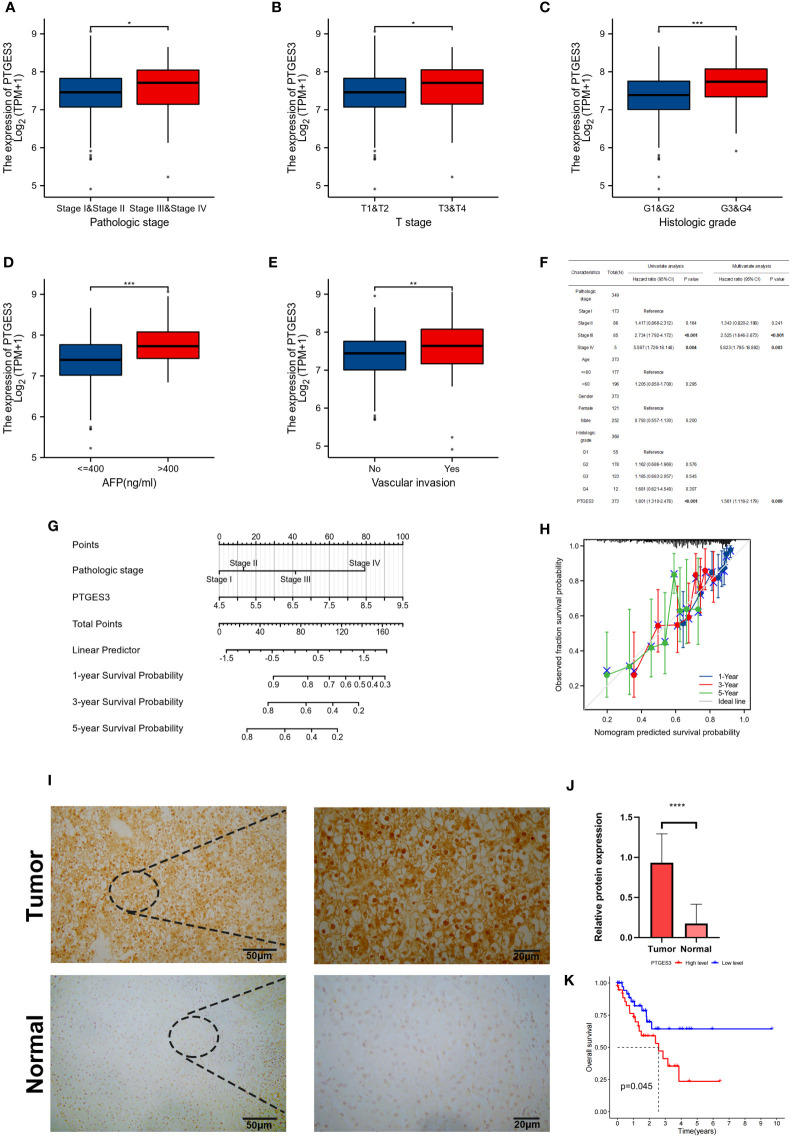
Correlation between PTGES3 expression and clinicopathologic features in HCC. **(A–E)** Correlation between PTGES3 expression and the pathological stage, histological grade, clinical T stage, alpha-fetoprotein (AFP) level, and vascular invasion in HCC. **(F)** Prognostic significance of PTGES3 in hepatocellular carcinoma was analyzed by univariate and multivariate Cox analyses. **(G)** Nomogram based on PTGES3 expression and pathological staging. **(H)** Correction analysis diagram of the nomogram. **(I)** Representative images of PTGES3 expression in the HCC tissues and adjacent normal liver tissues, analyzed by immunohistochemistry; original magnifications: ×40 and ×200. Scale bars: 50 μm. **(J)** Quantitative analysis of PTGES3 expression in HCC tissues based on mean density of immunohistochemical staining. **(K)** Survival analysis of PTGES3 expression in the collected samples. HCC, hepatocellular carcinoma; *p < 0.05; **p < 0.01; ***p < 0.001; and ****p < 0.0001.

In order to further explore the function of PTGES3 in the progression of HCC, we analyzed the association between PTGES3 expression and clinical phenotypes in hepatocellular carcinoma samples from TCGA. A higher PTGES3 expression was found in the higher pathologic stage, histologic grade, T stage, alpha-fetoprotein (AFP) level, and vascular invasion (**Figures 5A–E**). The univariate and multivariate Cox regression analyses were also selected to evaluate the impact of PTGES3 in HCC; the results revealed that PTGES3 was an independent predictive factor in HCC (**Figure 5F**). Additionally, we built a nomogram using PTGES3 expression and the pathologic stage to support the application of PTGES3 in HCC clinical evaluation (**Figure 5G**). The calibration curve was also made to evaluate the model’s accuracy for the prognostic assessment of HCC patients after 1, 3, and 5 years (**Figure 5H**). The ideal accuracy was found in our nomogram according to the results.

The protein expression level of PTGES3 was observed in HCC and paired normal tissues using immunohistochemistry, and our results demonstrated a higher protein expression level of PTGES3 in hepatocellular carcinoma tissues (**Figures 5I, J**). Subsequently, we collected the clinical data from these samples and explored the correlation between the PTGES3 protein expression level and clinical characteristics, from which we only found that PTGES3 expression correlated with the pathological tumor stage (**Table 1**). Furthermore, we performed survival analysis using our clinical data, and the results demonstrated that patients with a higher PTGES3 level led to a poor survival, which was consistent with our results from the online database (**Figure 5K**). Our results suggested that higher PTGES3 expression could be associated with malignant tumor development and worse prognosis in HCC patients.

**Table 1:** Clinical and pathological features of HCC patients.

Factor	Total number	PTGES3 expression high/low		*p*-value
Gender				
Male	65	47/18		
Female	15	12/3		0.542
Age (years)				
≤60	46	35/11		
>60	34	24/10		0.446
AFP (ng/ml)				
≤20	44	30/14		
>20	36	29/7		0.211
ALT (U/L)				
≤40	56	41/15		
>40	24	17/7		0.698
Pathologic stage				
I–II	45	29/16		
III–IV	35	30/5		0.032*
TNM stage				
I	39	26/13		
II	34	27/7		
III	7	6/1		0.308
Tumor size (cm)				
≤5	54	38/16		
>5	26	21/5		0.322
Microvascular invasion*				
No	43	30/13		
Yes	37	29/8		0.383
Hepatitis B status				
Negative	14	8/6		
Positive	66	51/15		0.12

AFP, α-fetoprotein; PTGES3 expression high: the expression of PTGES3 in HCC tissues was higher than the mean expressions in IHC; PTGES3 low expression: the expression of PTGES3 in HCC tissues was lower than the mean expressions in IHC. *Data are missing for some patients. *p < 0.05.

### 3.8 Gene enrichment analysis of PTGES3 in LIHC

**Figure 6:**
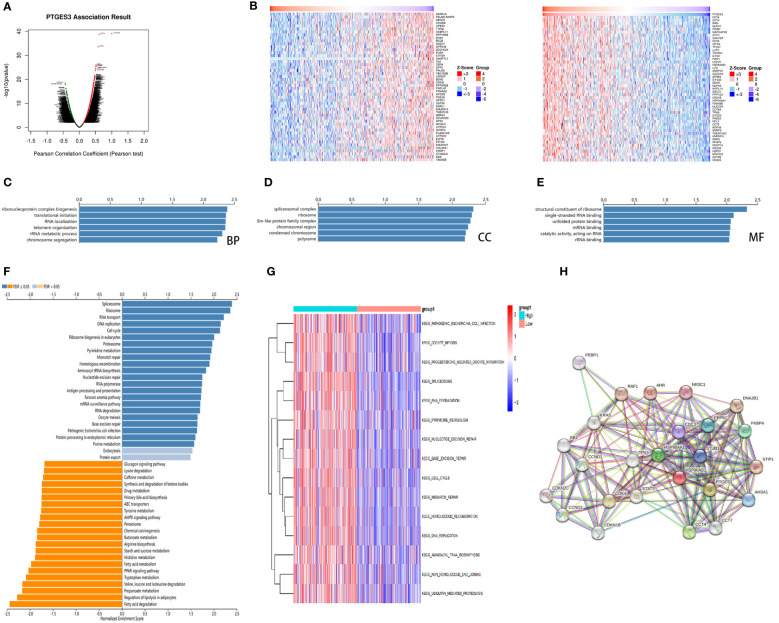
Gene enrichment analysis of PTGES3 in HCC. **(A)** Volcano figure of genes highly correlated with PTGES3 identified by Pearson test in the HCC cohort. **(B)** Heatmaps showing top 50 genes negatively and positively correlated with PTGES3 in HCC. The most enriched pathways of PTGES3 from GO analysis in **(C)** biological process (BP), **(D)** cell component (CC), and **(E)** molecular function (MF). **(F)** The enriched pathways of PTGES3 from KEGG analysis **(G)** The most enriched pathways of PTGES3 from GSVA. HCC, hepatocellular carcinoma. **(H)** The potential interaction network of PTGES3 was created using the STRING database.

For further analysis of PTGES3 in the regulation of HCC progression, we obtained the genes co-expressed with PTGES3 in HCC via the LinkedOmics database. Volcano plots depicted genes correlated with PTGES3, and the heatmaps demonstrated 50 positive and negatively correlated differentially co-expressed genes (**Figures 6A, B**). The KEGG and GO enrichment analyses were then performed to explore the pathways in which PTGES3 was involved in HCC progression. Biological progression results from GO enrichment analysis revealed dense chromosomes, ribonucleoprotein complex biogenesis, translation initiation, RNA localization, and telomere organization as the most enriched categories (**Figure 6C**). Spliceosome complexes, ribosomes, sm-like protein family complexes, chromosomal regions, and rRNA metabolic processes were significantly enriched in cellular components (**Figure 6D**). As regards, molecular function, single-stranded RNA binding, unfolded protein binding and catalytic activity, acting on RNA, structural constituent of ribosome, and mRNA binding were mainly enriched (**Figure 6E**). The results of KEGG analysis showed a significant enrichment of co-expressed genes in spliceosome, ribosome, RNA transport, DNA replication, and cell cycle pathways (**Figure 6F**). GSVA was performed to further investigate the biological influence of PTGES3 in HCC. We presented the top 15 pathways that were significantly correlated with PTGES3, which included RNA degradation, mismatch repair, and other pathways similar to our KEGG results (**Figure 6G**). After that, we constructed the protein–protein interaction network to speculate the potential interaction between PTGES3 and its related genes via the String database (**Figure 6H**). Our results revealed that PTGES3 expression was substantially correlated with metabolism-related pathways, such as cell cycle, mismatch repair, and spliceosome.

## 4 Discussion

PTGES3 is also regarded as p23, and its expressed co-chaperone protein p23 has an important regulating function interacting with heat shock proteins 90 (HSP90) as the substrate release factors (15). Upregulation of HSP90 is considered to have a significant influence on various cellular processes and tumor pathogenesis, predicting the poor prognosis of patients with malignant tumor such as HCC (16). Moreover, the aryl hydrocarbon receptor (AHR) in human cells is protected from degradation by p23 and Hsp90 interaction (17). AHR is involved in the occurrence of cancer as a ligand-activated signaling molecule through diverse signal pathways (18). In addition, p23 has been shown to possess other functions, such as interaction with p53 independent of Hsp90 (19). A study demonstrated that elevated p23 enhanced cell motility, which correlated with poor prognosis and a reduction in disease-free survival time in breast cancer patients (20). P23 has also been reported in the development of prostate cancer with anti-apoptotic capacity in malignant cells (21). However, biological information analysis for PTGES3 in pan-cancer has not been performed yet.

It has been reported that elevated PTGES3 expression was observed at BRCA, leading to poor survival according to previous results (22). According to our study, significant differences in PTGES3 expression level were found with 14 types of cancer. Interestingly, a downregulated expression level of PTGES3 was found in KICH. The results of TIMER analysis demonstrated a correlation between PTGES3 expression and clinical features such as tumor stage and age, which was significantly different in LIHC, KIRP, LUSC, and BRCA-lumb. Our Kaplan–Meier survival analysis and the Cox proportional hazards model analysis demonstrated that the upregulated expression of PTGES3 would lead to the poor prognosis in LIHC, ESCA, MESO, and LUAD. In contrast, higher PTGES3 expression was related to favorable prognosis in COAD and OV patients. Another research found that PTGES3 was highly expressed in COAD but was not associated with prognosis, which may be due to the different source/database (23). These results suggested that PTGES3 had a significant function in the indicated tumor types with tumor pathogenesis and prognostic prediction.

In addition, we deeply investigated the role of DNA methylation, CNV, and PTGES3 expression through GSCA. Methylation in liver cancer progression could activate the expression of proto-oncogene promoting tumor progression and deteriorate the condition of HCC patients (24). PTGES3 expression was closely related to CNV and DNA methylation. In the era of precision medicine, TMB has influenced the development of immunotherapy and has become a promising pan-cancer prediction biomarker with a significant reference value (25, 26). Similarly, MSI also served as a prognostic biomarker for immune checkpoint inhibitors (ICIs) (27). Our study indicated that PTGES3 expression has influenced TMB in 12 types of cancer and MSI in 10 types of cancer, which affected the response to immune checkpoint inhibitory therapy. PTGES3 expression was also found to be significantly positively correlated with MMR gene expression in most tumors.

Exploring the connection of the tumor immune microenvironment (TME) and tumor cells was important for the removal of tumor cells and immune escape (28). In a wide range of cancer, our investigation found that PTGES3 expression has substantial relationships with the infiltration degree of CD4^+^ T cells and MDSCs. Th2 cells were known to suppress the development of Th1 cells and the release of IFN-γ, but they also secreted IL-4 and IL-10, which stimulated the growth of tumor cells (29). The strong correlation between PTGES3 and these different immune cell types suggests that PTGES3 affected the polarization of Th2 and macrophage cells, which, in turn, promoted the tumor development. Immune checkpoints were crucial immunomodulators for maintaining immunological homeostasis and avoiding autoimmunity, which consisted of inhibitory and stimulant pathways that were essential for regulating the kind, intensity, and duration of immune responses (30). P23 exhibited cytosolic PTGES3 activity, where it increased in peritoneal macrophages reacting with lipopolysaccharide (LPS) (31). It has been found that p23 deficiency increased the phagocytic activity of LPS-induced macrophages, which also stimulated macrophage migration via KIF15 instability (32). Additionally, we discovered a favorable correlation between PTGES3 expression and genes relevant to immunological checkpoints, which included PD-L1, TIGIT, and HAVCR2, especially in HCC, which confirmed the critical role of PTGES3 in liver cancer though regulating tumor immunity and macrophage polarization. These studies suggest that upregulated PTGES3 expression could play a role in the immunosuppressive microenvironment, providing potential immunotherapy targets in cancer patients.

KEGG and GSVA results revealed that the co-expressed genes of PTGES3 were mainly enriched in processes such as spliceosome, DNA replication, and mismatch repair and downregulated in the regulation of lipolization in adipocytes and fatty acid degradation. Deregulation of cell cycle contributed to the mutation of protein, which was closely associated with harmful cell processes and carcinogenesis (33). Another research demonstrated that PTGES3 might be a predictive prognostic biomarker correlating with DNA regulation and immune infiltrates in LUAD (34). Furthermore, our analysis validated the association between the PTGES3 expression and the pathological stage through our collected clinical data, which were further integrated to build a nomogram for the application of PTGES3 in HCC prognostic assessment. According to our drug analysis, PTGES3 expression was sensitive to 5-fluorouracil (5-Fu) and Sorafenib, which was currently applied in HCC therapy (35). Gedunin is a plant product that can bind p23, thus blocking its function and leading to programmed cell death in malignant cells (36). It has been reported that AIL, a quassinoid natural product, blocked the tumor growth and metastasis of castration-resistant prostate cancer (CRPC) and was regarded as a potential candidate for the treatment of CRPC (37). Another research also reported that AIL inhibited tumor growth in GC cells through downregulating the DNA repair activity of malignant cells, which suggest that PTGES3 has the potential as a therapy target in novel treatments (38). It has been demonstrated that leukemia cells with PTGES3 knockdown had an elevated therapeutic response in bone marrow (39). These results suggested that PTGES3 was involved in tumor oncogenesis through gene alterations and tumor mutation, which had the potential to become a target in individual tumor therapy. Our work still had some limitations. Most of our data were obtained from online databases, which might lead to analytic bias. The detailed mechanisms on how PTGES3 participates in immune dysfunction and HCC oncogenesis remain unknown. Information on the specific pathways of PTGES3 involved in such processes still needs to be investigated. Further analysis should be performed to explore the function of PTGES3 in HCC development.

## 5 Conclusion

In conclusion, PTGES3 expression in tumor was statistically higher than normal tissues both at the transcriptional and protein levels, which was also linked to their prognosis. PTGES3 expression was also associated with the abundance of immune cell infiltration, the response to ICIs, and multiple pathways related to tumor progression in HCC. Therefore, PTGES3 was suggested as a cancer-promoting biomarker in HCC. Given that multifactorial mechanisms such as immune escape make it difficult to improve the prognosis of HCC, PTGES3 might contribute to HCC targeting therapies in the future.

## Data availability statement

The original contributions presented in the study are included in the article/**Supplementary Material**. Further inquiries can be directed to the corresponding author.

## Ethics statement

The studies involving human participants were reviewed and approved by Affiliated Hospital of Qingdao University. The patients/participants provided their written informed consent to participate in this study.

## Author contributions

HW was involved in the data analysis and drafting most of the manuscript. HW and PS took part in the design and data collection process of the study. RYY and WRZ conducted the experiments. PS and JY edited and reviewed the paper. XSZ and KH took part in the manuscript revision and offered substantial help. All authors contributed to the article and approved the submitted version.

## Supplementary material

Supplementary Table 1 The number of each type of tumor in this study from TCGA.
